# Unmet Needs of Families of Adults With Intellectual and Development Disabilities on Waiting Lists

**DOI:** 10.1093/geroni/igaa057.2413

**Published:** 2020-12-16

**Authors:** Tamar Heller

**Affiliations:** University of Illinois, Chicago, Illinois, United States

## Abstract

Most adults with intellectual and developmental disabilities live at home with their aging parents. Given the large waiting lists for residential and home-based services, families face many unmet service and support needs. The author will present results of a study that examined the impact of a Medicaid waiver program that provided either home-based or residential placements to 444 families of adults with IDD who were living at home at baseline through surveys at baseline and two years later. Families who did not receive the waiver services still had high unmet needs for person-centered planning training, networking with other families, respite, advocacy services, assistive technology, and home modifications at follow up. Regardless of services received, class members from minority backgrounds had more unmet needs than white class members, indicating the need for more targeted efforts to reach minority families.

